# Climate and forest loss interactively restructure trait composition across a human‐modified landscape

**DOI:** 10.1002/ece3.9361

**Published:** 2022-10-30

**Authors:** Meghna Krishnadas

**Affiliations:** ^1^ CSIR‐ Centre for Cellular and Molecular Biology Hyderabad Telangana India

**Keywords:** fragments, human‐modified forest, plant community, trait covariance, trait–climate

## Abstract

Traits determine species response to climate conditions and the match between phenotypes and climate mediates spatial variation in species composition. These trait–climate linkages can be disrupted in human‐modified landscapes. Human land use creates forest fragments where dispersal limitation or edge effects exclude species that may otherwise suit a given macroclimate. Furthermore, stressful macroclimate can limit viable trait combinations such that only a subset of values of any given trait occurs with respect to another trait, resulting in stronger trait covariance. Because forest loss can compound climatic stress, trait covariance from benign to harsher climates is expected to be stronger in fragments compared to contiguous forests. In a wet tropical forest landscape in the Western Ghats Biodiversity Hotspot of peninsular India, I compared fragments with adjacent contiguous forests for signatures of trait‐mediated assembly of tree communities. Using four key plant traits—seed size, specific leaf area (SLA), wood density, and maximum height—I evaluated trait–abundance associations and trait covariance across climate, soil, and elevation gradients. In the contiguous forest, smaller‐seeded, shorter, thinner‐leaved species became more abundant from low to high elevations. In fragments, species with higher SLA were more abundant at sites with more seasonal climates and lower precipitation, and larger seeded species were less abundant at warmer sites. However, traits only weakly predicted abundances in both habitats. Moreover, only contiguous forests exhibited significant compositional change via traits, driven by trait syndromes varying along a composite gradient defined by elevation, water deficit, and soil C:N ratio. Site‐level trait covariance revealed that warmer, wetter conditions in fragments favored taller species for given seed size, as compared to similar conditions in contiguous forests. Overall, trait syndromes and trait covariance, rather than single traits, determined the phenotypes best suited to macroclimate conditions and should inform management or restoration goals in fragments.

## Graphical abstract

Habitat loss can alter trait‐mediated assembly across climate gradients. In a human‐modified forest landscape, traits explained composition across a composite gradient of elevation and soil C:N ratio in contiguous forests, but did not explain the compositional change in fragments. Trait covariance revealed divergent constraints on phenotypes in fragments vs. contiguous forests.

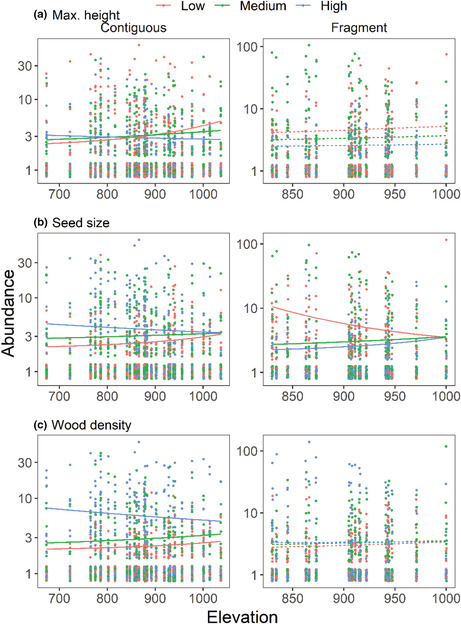

## 1 INTRODUCTION

Functional traits influence community assembly by mediating species' performance in different climate conditions (Adler et al., 2013; Paine et al., 2011; Poorter et al., 2008; Sonnier et al., 2010). Trait–climate linkages influence species distributions, changes in species composition, and the response of ecological communities to anthropogenic change (Bernard‐Verdier et al., 2012; Cornwell & Ackerly, 2009; Harrison & LaForgia, 2019; Méndez‐Toribio et al., 2020; Poorter et al., 2019; Venail et al., 2015). In human‐modified forest landscapes, remnant habitats exist as fragments embedded within a matrix of other land uses (Barlow et al., 2007; Melo et al., 2013). Forest fragments experience edge effects, e.g., increased light, ambient temperature, and lower soil moisture, that make the microclimate warmer and drier than interior conditions of contiguous forest (De Frenne et al., 2019, 2015; Zellweger et al., 2020). Loss of forest cover can also enhance climatic stress in a landscape (Arroyo‐Rodríguez et al., 2017; Laurance, 2004). These changes to the macro‐ and microclimate can shift the role of traits in mediating community assembly in contiguous forests vs. fragments across a landscape (Fernandes Neto et al., 2019; Krishnadas, Beckman, et al., 2018; Lebrija‐Trejos et al., 2010; Poorter et al., 2019; Zirbel & Brudvig, 2020).

The role of macroclimate in shaping communities within fragments compared to contiguous forests should be reflected in the link between traits and species abundances (trait–abundance associations) across climate gradients in each habitat. For trees, four traits explain substantial variation in plant performance (Wieczynski et al., 2019), which can govern their response to macroclimate stress. Seed size reflects the tolerance–fecundity trade‐off (Muller‐Landau, 2010) with larger seeded species doing better in stressful conditions, e.g., shade or drought (Baraloto & Forget, 2007; Bruun & ten Brink, 2008; Westoby et al., 2002). Wood density and specific leaf area (SLA) mediate differences in species distributions across gradients of rainfall and seasonality (Krishnadas et al., 2021; Moles et al., 2014). Maximum height can influence species' performance in response to light and water availability—competition for light in shaded conditions favors taller species, whereas shorter species are less vulnerable to hydraulic failure in dry conditions (Méndez‐Toribio et al., 2017; Rüger et al., 2012; Tyree, 2003).

In tropical forests, understory light availability decreases in wetter macroclimates resulting in larger seeds being more successful at seedling establishment and survival (Bruun & ten Brink, 2008). Deeper shade can limit photosynthesis in the forest understory (Muscarella et al., 2016; Poorter et al., 2019) to advantage species with denser wood or lower SLA whose conservative resource‐use strategies allow them to withstand light‐limited conditions in wetter sites (Poorter et al., 2008; Wright et al., 2010). Within fragments, however, greater light availability could tilt the competitive advantage toward species with smaller seeds, lighter wood, and higher SLA even in wet sites (Krishnadas et al., 2020; Osuri & Sankaran, 2016a). Alternatively, resource conservative strategies of species with denser wood and lower SLA may help cope with increasing water deficit (Muscarella et al., 2016; O'Brien et al., 2017; Sterck et al., 2006). This correlation between water deficit and lower SLA or higher wood density may be stronger in fragments where water deficit can intensify compared to contiguous forests.

In addition, climate can vary considerably over relatively small geographic distances across elevation gradients. Associated changes in tree community composition (Krishnadas & Osuri, 2020) can occur via trait‐mediated responses (Butterfield & Suding, 2013; Chalmandrier et al., 2015; Swenson et al., 2011). Compositional variation across the elevation gradient can be homogenized by forest loss and land‐use change (Frishkoff et al., 2019; Krishnadas & Osuri, 2020; Yano et al., 2021). Forest loss can make the landscape warmer and drier (Laurance, 2004) and fragments can be less buffered from macroclimate stress than contiguous forests (Arroyo‐Rodríguez et al., 2017; Frey et al., 2016). If microclimates within fragments modulate or override the effects of macroclimatic gradients (Arroyo‐Rodríguez et al., 2017; Davis et al., 2019; De Frenne et al., 2019), it will obscure trait–abundance associations seen across macroclimate gradients in contiguous forests.

Response to macroclimate gradients can occur via the coordinated selection of multiple traits, rather than individual traits (Dwyer & Laughlin, 2017; Laughlin & Messier, 2015). Specifically, climatic conditions can impose trade‐offs that constrain trait combinations, quantified as trait covariance within communities (Dwyer & Laughlin, 2017). Stronger site‐level trait covariance suggests that climate stress curtails viable trait combinations in locally co‐occurring species, resulting in fewer viable phenotypes than what is possible in less stressful conditions (Dwyer & Laughlin, 2017; Umaña & Swenson, 2018). Moreover, positive covariance (e.g., large seeded and dense wooded, or large seeded and tall) suggests coordinated selection for extreme trait values (Shen et al., 2019; Umaña & Swenson, 2018). Negative covariance may imply selection pressure by multiple factors on traits for a given climate. As an example, drier macroclimates favor thicker leaves and higher light availability at these sites may concomitantly increase the recruitment of smaller‐seeded species (Muscarella et al., 2016; Tyree, 2003).

Since forest cover tends to buffer the impact of unsuitable macroclimate (De Frenne et al., 2019), trees in fragments may experience greater stress than contiguous forests in harsher climates (Arroyo‐Rodríguez et al., 2017; Didham & Lawton, 1999; Krishnadas, Bagchi, et al., 2018). As a result, constraints on viable trait combinations across a macroclimate gradient can be tighter in fragments compared to contiguous forests, especially as macroclimate stress increases. Alternatively, macroclimate influence on trait covariance could weaken in fragments if similar phenotypes prevail everywhere across the climate gradient. For instance, heterogeneous light levels in fragments may promote the recruitment of smaller‐seeded species, allowing a wider range of viable combinations of seed size with SLA or wood density regardless of macroclimate. In contiguous forests by comparison, increasing shade in wetter sites could restrict trait combinations to shade‐tolerant phenotypes characterized by combinations of large seeds and denser wood or thicker leaves.

Here, I assessed how the macroclimate shapes the assembly of tree communities in fragments vs. contiguous forests. Across a human‐modified, wet tropical landscape in peninsular India, I quantified trait‐mediated distributions of tree species and constraints on trait combinations across macroclimate gradients. Specifically, I examined whether:

1. Fragmented and contiguous forests differ in trait‐mediated species distributions across macroclimate gradients.
2. Across climate gradients, compositional variation of contiguous forests and fragments is shaped by individual traits or multi‐trait phenotypes.
3. Increasing macroclimate stress constraints trait combinations more strongly in fragments compared to contiguous forests.

## 2 MATERIALS AND METHODS

### 2.1 Study area

The study was conducted in the Western Ghats biodiversity hotspot of southern India. The landscape (12.17°N, 75.80°E), located in Kodagu district of Karnataka state, is classified as mid‐elevation evergreen forests (Pascal, 1986). Elevations ranged between 673 and 1037 m ASL in contiguous forests and 830–1030 m ASL in fragments. Mean annual precipitation ranged from 2400 to 3800 mm across sites. The dry season varies from 6 to 8 months when moving from west to east. The region has well‐drained clayey and loamy type soils, with soil types remaining broadly similar across the region (Anonymous, 1998). The sampled areas were fragmented ca. 100 years ago with the expansion of coffee plantations, paddy fields, and human settlements. For the last 40 years, both contiguous and fragmented forests were legally protected against land‐use change, timber extraction, and hunting. Local communities also protect the fragments as sacred groves (Bhagwat et al., 2005). However, the expansion of plantations occurred as recently as 2000 AD, driven by land encroachment in response to global demand for coffee (Ambinakudige & Choi, 2009). While many smaller fragments have disappeared and forest edges have degraded (Osuri et al., 2014), undisturbed areas were selected for the study based on local consultation and field surveys of disturbance signs. Refer to Krishnadas and Osuri (2020) for a map of the study area and plot locations.

### 2.2 Data collection

#### 2.2.1 Vegetation sampling

Data on tree communities were obtained from an online repository (Osuri & Sankaran, 2016b). The dataset comprised 50 square plots of 30 × 30 m, with 31 plots at eight locations within contiguous forests and 19 plots in eight forest fragments. All plots were censused during Jan–Dec 2011. Sampled fragments ranged from 5 to 10 ha in size, chosen to avoid ongoing or recent disturbance or human use. Vegetation plots were placed at least 50 m from fragment edges and 50 m apart from each other. The contiguous forest plots were located within a complex of structurally connected nature reserves adjacent to the fragments. Shade coffee plantations dominated the land use in the human‐modified matrix. For more details on the plot locations and placement, see Osuri and Sankaran (2016b). Species were identified using field keys, regional floras (Pascal & Ramesh, 1997), and with input from experienced botanists. About 3% of individuals were excluded as they had no leaves or the canopy was obscured by climbers and remained unidentified.

#### 2.2.2 Traits

I used the four functional traits—seed size, wood density, specific leaf area (SLA), and maximum height. Trait data were available from the Supporting Information included with Osuri and Sankaran (2016b) and had been collected between November 2011 and January 2013 following standard protocols (Pérez‐Harguindeguy et al., 2013) from trees within or adjacent to the vegetation plots. The data collection methods have been detailed in Osuri and Sankaran (2016b). Here, I briefly repeat the main.

For SLA, five mature, healthy, sun‐exposed canopy leaves were collected per tree at the end of the wet season (October–December) for 358 trees comprising 79 species. Leaf areas were estimated using the black spot leaf area calculator (Varma & Osuri, 2013), after which leaves were oven‐dried at 60°C for 72 h to obtain dry weights. Wood density was estimated by dividing dry weight by fresh volume of trunk wood cores collected with an increment borer for 352 trees representing 74 species. Thirty‐six species for which adequate primary data could not be collected, wood density was collated from secondary sources (Chave et al., 2009). Seed size was quantified as the length of the primary seed axis for 34 species (1879 seeds in total). Additionally, seed size data were obtained from two other mid‐elevation evergreen forests in the Western Ghats (D. Mudappa, unpublished data). For species without primary measurements, seed lengths were collated from published secondary sources. Maximum heights of species were obtained in the field and from secondary sources. For details of secondary sources, please see Osuri and Sankaran (2016a).

#### 2.2.3 Climate data

Climatological water deficit (Chave et al., 2014), henceforth CWD, was used to quantify site‐level variation in seasonal water stress (Condit et al., 2013; Vicente‐Serrano et al., 2013). CWD, measured in mm/year, is always a negative number since it is the difference between rainfall and evapotranspiration during dry months only, and more negative values indicate greater water deficit in the dry season, i.e., an index of seasonality. CWD data were downloaded from the source cited in Chave et al. (2014; http://chave.ups‐tlse.fr/pantropical_allometry.htm#CWD). Site‐level climate aridity was characterized by annual evapotranspiration data, available at a spatial resolution of 1 km^2^, and can be downloaded from the CGIAR‐CSI GeoPortal (https://cgiarcsi.community). Gradients in precipitation and temperature were characterized with data from WorldClim, collated per 1 km^2^ (Hijmans et al., 2005). I used mean annual precipitation, mean precipitation of the driest quarter, mean precipitation of the warmest quarter, mean annual temperature, mean temperature of the warmest quarter, and the mean temperature of the driest quarter.

### 2.3 Statistical analysis

All analyses were conducted in program R version 3.6.3 (R Development Core Team, 2019) using relevant packages as detailed below in the different sections.

#### 2.3.1 Trait–climate interactions

Interactions of traits with different climate variables explaining species abundances in contiguous forests and fragments were assessed using generalized linear mixed‐effects models (Brown et al., 2014; Jamil et al., 2013), implemented using package glmmTMB (Brooks et al., 2017). As detailed in Jamil et al. (2013), the response variable was the abundance of each species per site, including zeros, modeled using a negative binomial error structure. Site‐to‐site variation in abundance was modeled in relation to an interaction between traits and macroclimate variables, separately for fragments and contiguous forests. Plots (nested within sites) and species were included as random intercepts. A zero‐inflation component was included to account for the fact that most species were absent in many plots. Zero inflation was modeled for the intercept and as a function of the trait in each model. Zero‐inflated models consistently performed better than models without zero inflation (based on AIC).

I used principal components analysis to decompose CWD, aridity, and the temperature and precipitation variables from WorldClim into three composite axes that together explained ~95% of the variation in climate across the entire landscape (Figure S1). Climate axis 1 explained 63.2% variation and primarily captured the precipitation gradient, with positive values corresponding with greater precipitation. Climate axis 2 captured 21.1% of macroclimate variation and represented a temperature gradient; more positive values indicated warmer sites. Climate axis 3 captured the seasonality gradient represented by CWD (11.3% variation), with positive values of the axis corresponding with lower seasonality. Because climate variables were correlated in both contiguous forests (Figure S2) and fragments (Figure S3), I used the variance inflation factor to check multicollinearity among variables in all models (Fox & Weisberg, 2019), using the criteria of removing variables with VIF > 4. Separate models were run per trait. In all models, Climate axis 1 (precipitation gradient), Climate axis 2 (temperature gradient), Climate axis 3 (seasonality gradient), and soil C:N ratio were the final climate predictors retained. Elevation was correlated with the precipitation gradient and temperature gradient, and was previously found to be a key predictor of compositional change in this landscape (Krishnadas & Osuri, 2020). So, I also tested models with elevation as the sole predictor of changes in trait–abundance associations.

#### 2.3.2 RLQ and fourth‐corner analyses

The second question was to assess trait‐mediated changes in species composition across climate gradients. This may occur via (a) individual traits changing along individual climate gradients, (b) individual traits changing along a combination of climate conditions, or (c) trait combinations (functional syndromes) responding to a combination of climate conditions. These possibilities were evaluated using the RLQ and fourth‐corner methods. RLQ identifies the links between climate gradients and trait syndromes as reflected by species abundances, and fourth‐corner analysis performs formal statistical tests of hypotheses regarding trait–climate linkages (Dray et al., 2014; Peres‐Neto et al., 2017). For this procedure, R is the matrix of climate variables by sample sites, Q has Species × Trait data, and L is a Site × Species matrix of abundances or occurrences. The RLQ outputs decompose correlations in the data along orthogonal axes for a global measure of trait–climate linkages (Dray et al., 2014). The analysis tests for two relationships using randomization tests. First, the environment influences the distribution of species and next, traits influence the composition of species assemblages found in samples with given environmental conditions. Trait–environment associations can be considered valid only if both models are significant. Finally, the fourth‐corner analysis evaluates multiple bivariate correlations between traits and climate variables, using permutation tests on the RLQ outputs to assess the relationship between (a) individual functional traits and single or combined climate gradients (RLQ climate scores), and (b) climate variables and functional syndromes (RLQ traits scores; Dray et al., 2014).

Following the procedure detailed by Dray et al. (2014), I first analyzed the L table using correspondence analysis (CA), followed by standardized principal components analysis (PCA) of the R and Q tables. Then, I evaluated the proportions of variation in the climate ordination and the trait ordination from the RLQ axes. I tested the global significance of trait–climate relationships (RLQ axes) and bivariate correlations between traits and climate variables with the “randtest.rlq” and “fourthcorner.rlq” functions, respectively. To control false‐positive error rates in multiple comparisons, I used the Benjamini–Hochberg false discovery rate procedure (Benjamini & Hochberg, 1995) with 9999 permutations to estimate *p*‐values.

I ran separate RLQ analyses for fragments and contiguous forests to compare their trait–climate linkages. SLA and seed size were log transformed and all traits were scaled by their mean and standard deviation prior to analysis. All calculations and graphs were made using package “ade4” in R version 4.0.1 (R Core Team, 2017). For this analysis, I included all climate variables together since the multivariate analysis accounted for collinearity among variables.

#### 2.3.3 Trait covariance

To assess how climate gradients influenced trait covariance, for each plot I calculated each pairwise covariance of the four traits using methods and R codes in Dwyer and Laughlin (2017). I used linear models to model plot‐level covariance of each trait pair as a function of the interactions of forest type (fragment/contiguous) with macroclimate conditions of a plot. Preliminary analysis with mixed‐effects models showed minimal effects of site‐level variance on model coefficients and variance explained, and higher AIC than linear models. As with trait–abundance associations, correlated predictors were removed using VIF to reduce collinearity. Because elevation correlated strongly with multiple variables, changes in site‐level trait covariance with elevation were tested in separate models.

## 3 RESULTS

### 3.1 Trait–abundance associations

**FIGURE 1:**
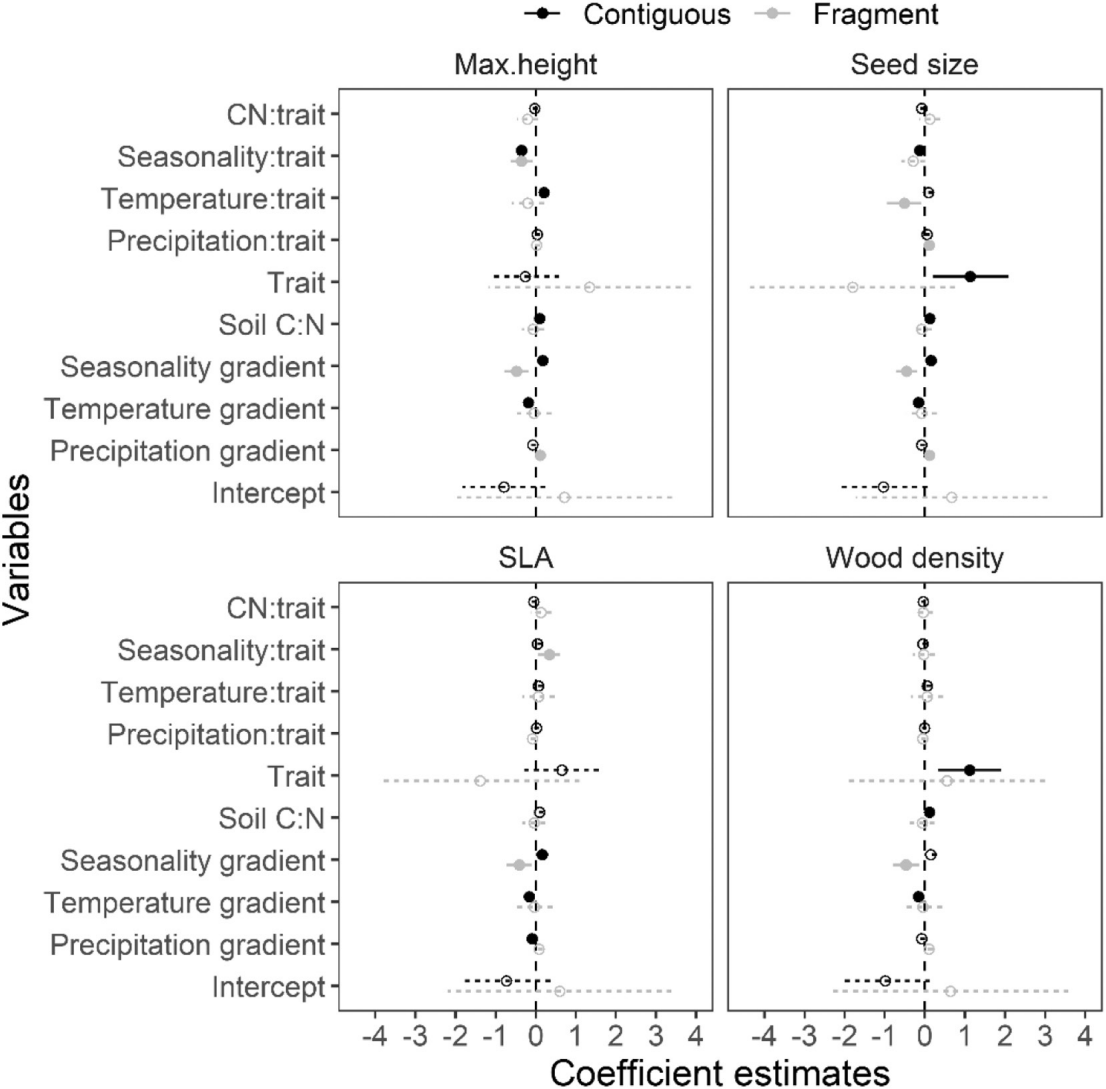
Coefficient estimates from trait–climate interaction models. Changes in species abundances across macroclimate gradients were modeled in relation to four functional traits for contiguous forests and fragments. Generalized linear mixed‐effects models with negative binomial errors and a zero‐inflation component were used to model the relationships. The climate across sites was characterized using climatic water deficit, soil C:N ratio, and two composite climate axes derived from principal components analysis of selected climate variables from WorldClim data (see section 2). Points depict coefficient estimates and error bars show associated confidence intervals. Solid lines and filled circles indicate significant relationships where the confidence interval does not overlap zero (vertical dashed line).

As expected, the four traits differed in their interaction with macroclimate variables to predict species distributions across the landscape, and trait–climate interactions differed between fragments and contiguous forests (Figure 1). In contiguous forests, lower water deficit (positive values of Climate PC axis 3) corresponded to increased abundance of shorter statured species (interaction coefficient, *β* = −0.34, *SE* = 0.06, *p*‐value < .01). This relationship remained similar in fragments (interaction coefficient, *β* = −0.36, *SE* = 0.06, *p*‐value < .001). Taller species increased in abundance at warmer sites in contiguous forests (interaction coefficient, *β* = 0.21, *SE* = 0.05, *p*‐value ≪ 0.001), but fragments had no interaction of height and temperature. While overall abundance increased with seed size in contiguous forests (*β* = 1.13, *SE* = 0.48, *p*‐value = .02), this trend reversed as seasonal water deficit decreased (interaction coefficient, *β* = −0.18, *SE* = 0.06, *p*‐value = .03). In fragments, larger‐seeded species declined at warmer sites along the temperature gradient (interaction coefficient, *β* = −0.56, *SE* = 0.21, *p*‐value < .01). All three climate gradients correlated with seed size–abundance associations in contiguous forests. Larger‐seeded species decreased at less rainy (*β* = 0.11, *SE* = 0.04, *p*‐value < .01), warmer (*β* = −0.51, *SE* = 0.22, *p*‐value = .02), and less seasonal sites (*β* = −0.29, *SE* = 0.15, *p*‐value = .05). Greater seasonal water deficit in fragments also increased abundances of high SLA species (*β* = 0.34, *SE* = 0.14, *p*‐value = .01). Wood density did not interact with any climate gradient to shape abundances in either contiguous forests or fragments.

**FIGURE 2:**
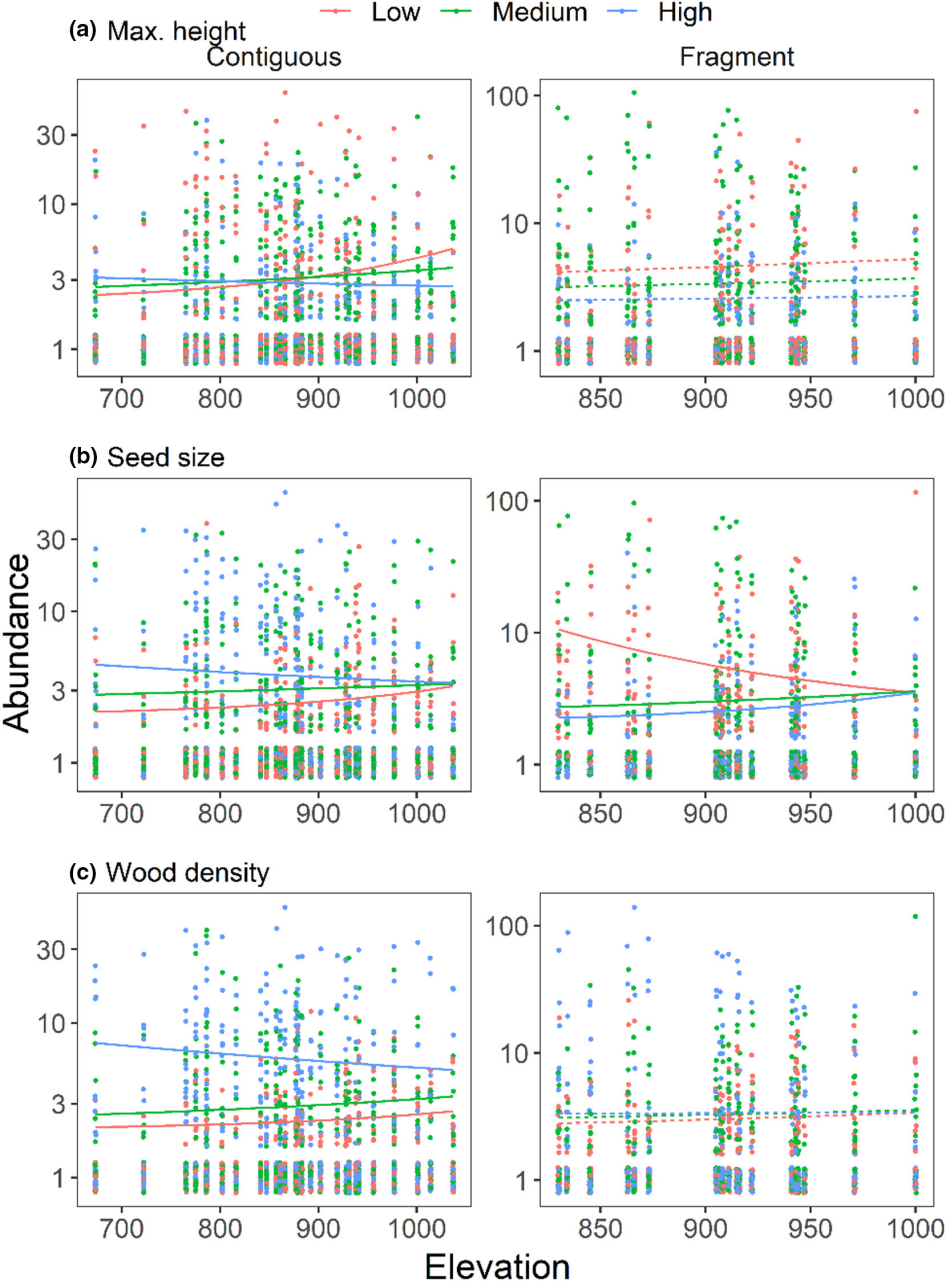
Trait–abundance association with elevation. Changes in species abundances across elevational gradients in contiguous forests and fragments were modeled in relation to four functional traits. Generalized linear mixed‐effects models with negative binomial errors and a zero‐inflation component were used to model the relationships. Points indicate observed values and lines show predictions at the 10th (low), 50th (medium), and 90th (high) percentiles of trait values. Note the log scale for the *Y* axis and differing ranges for contiguous forests and fragments. Solid and dashed lines, respectively, indicate significant and non‐significant trait–elevation interactions.

Models with elevation as the sole predictor explained similar extents of variation as models with multiple climate predictors in contiguous forests, but not fragments (Table S1). In contiguous forests, increase in elevation decreased abundances of species with taller stature (*β* = −0.003, *SE* = 0.0007, *p*‐value < .01), denser wood (*β* = −0.002, *SE* = 0.0006, *p*‐value < .01), and larger seeds (*β* = −0.002, *SE* = 0.0006, *p*‐value = .001). For every 100 m increase in elevation, there was a decrease of .009 m in species height, 0.1 g/cc in wood density, and 0.5 cm in seed size (Figure 2). Only seed size showed a significant interaction with elevation in fragments, with larger‐seeded species having higher abundance at higher elevations (Table S2).

Overall, trait interactions with climate/elevation at most explained ~11% of the variation in species abundances. The model with maximum height best explained the change in species abundance across macroclimate gradients (Table S1). Except for the model with maximum height, random effects of species explained more variation than fixed effects (Table S1, see conditional vs. marginal *R* ^2^), indicating that unmeasured species‐level factors contributed substantially to changes in species' abundances. Site‐level random effects explained minimal variation.

### 3.2 RLQ and fourth‐corner analysis

**FIGURE 3:**
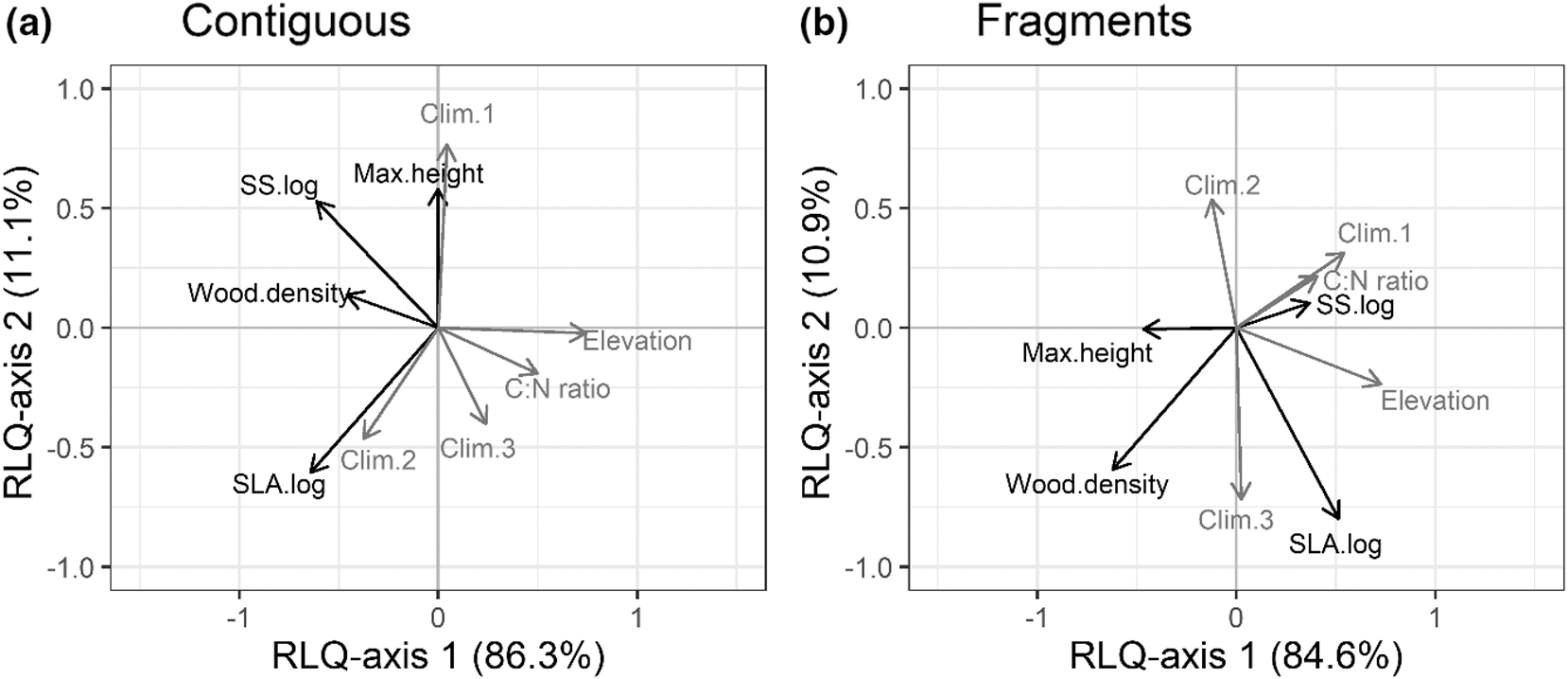
RLQ analysis of trait–climate linkages. For (a) contiguous forests and (b) forest fragments, correspondence analysis was used to assess links among species' traits, species composition, and gradients in macroclimate conditions. Climate was characterized using elevation, climatic water deficit (CWD), precipitation gradient (Clim.1, precipitation gradient), temperature gradient (Clim.2, temperature gradient), soil C:N ratio, and atmospheric aridity. Only contiguous forests showed significant relationships after correcting for multiple comparisons (see Figure 4).

Only contiguous forests had significant trait–climate linkages driving compositional change—the first RLQ axis explained 84.6% and the second axis 13.5% of the cross‐variance in traits and macroclimate (Figure 3a). Correlations of climate with composition (standardized *r* = 4.26, *p*‐value = .004) and traits with climate (standardized *r* = 2.09, *p*‐value = .04) remained statistically significant after randomization tests. In fragments, 88% of the cross‐variance between traits and climate was explained by the model (Figure 3b), but the results were not statistically significant after accounting for multiple comparisons.

**FIGURE 4:**
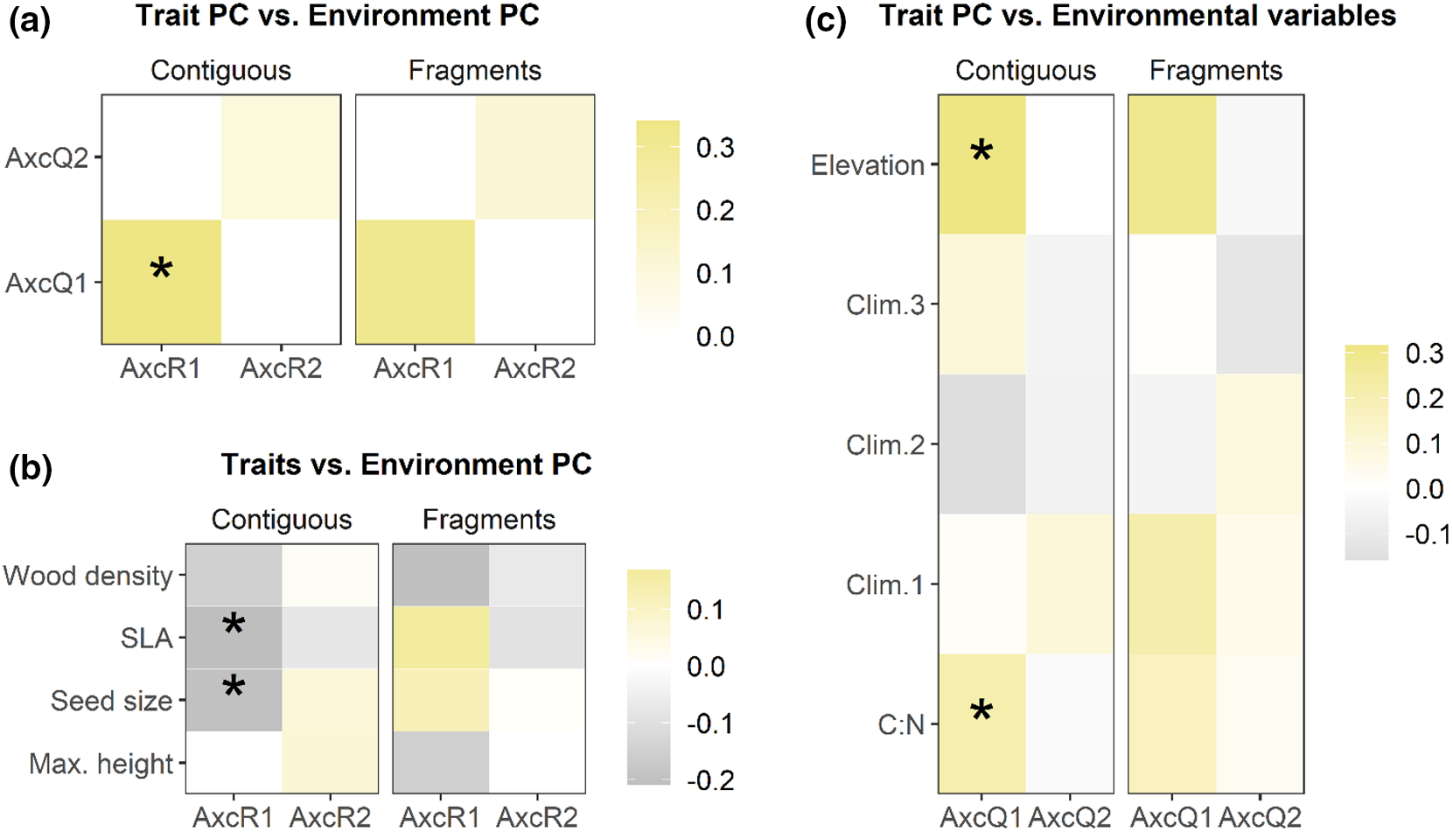
Fourth‐corner analysis of RLQ outputs. The trait‐mediated compositional change was assessed with respect to macroclimate gradients in fragments vs. contiguous forests, yielding relationships between (a) composite trait axes and composite macroclimate gradients, (b) individual traits and composite macroclimate gradients, and (c) individual traits and individual macroclimate variables. Cell color tending toward darker gray indicates more negative correlations and darker yellows indicate an increasingly positive correlation, with white indicating no correlation (see the scale in the figure). Asterisk indicates a statistically significant correlation (*p*‐value < .05). Climate was characterized using elevation, precipitation gradient (Clim.1), temperature gradient (Clim.2), seasonality gradient (Clim.3), and soil C:N ratio (C:N). *p*‐Values for the significance of relationships were assessed using Benjamini–Hochberg correction for false‐positive results through 9999 random permutations of matrices.

In contiguous forests, trait axis R1, representing a syndrome of lower values of wood density, SLA, and seed size, significantly increased along climate axis Q1 (higher elevations, lower water deficit), and higher C:N ratio (standardized *r* = 0.34, *p*‐value ≪ .001; Figure 4a). This pattern was mainly driven by changes in SLA (standardized *r* = −2.47, *p*‐value = .06) and seed size (standardized *r* = −2.34, *p*‐value = .09) across axis Q1 representing a gradient of higher elevation and greater C:N ratio (Figure 4b). The relationship between individual gradients and trait axes showed an increase in smaller‐seeded, lighter‐wooded, and thicker‐leaved species at higher elevations (standardized *r* = 4.04, *p*‐value < .001) and in soils with higher C:N ratio (standardized *r* = 2.73, *p*‐value = .04; Figure 4c). Fourth‐corner analysis for fragments revealed no significant correlations between trait axes and climate gradients. No individual trait showed a significant correlation with any individual climate variable in either fragments or contiguous forests.

### 3.3 Trait covariance

**FIGURE 5:**
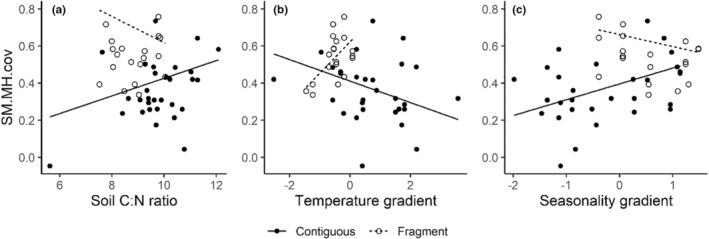
Trait covariance along climate gradients. Site‐level covariance of each pair of traits was modeled in relation to macroclimate gradients across contiguous forests and fragments using multiple linear regression. The climate across sites was characterized using soil C:N ratio and three composite climate axes derived from principal components analysis of selected climate variables from WorldClim data (see section 2). Only relationships with significant trends are shown here. See Table S3 for all coefficient estimates.

All coefficients for the models of trait covariance are provided in Table S3. Only seed size–maximum height (SS‐MH) covariance showed significant relationships with climate variables (Figure 5a–c). Within contiguous forests, SS‐MH covariance increased with greater C:N ratio (*β* = 0.04, *SE* = 0.02, *p*‐value = .02) and lower water deficit (*β* = 0.08, *SE* = 0.03, *p*‐value = .02), and decreased at warmer sites (*β* = −0.06, *SE* = 0.02, *p*‐value = .003). All relationships reversed in fragments, with larger effect sizes. Seasonal water deficit (*β* = −0.14, *SE* = 0.06, *p*‐value = .02) and temperature (*β* = 0.22, *SE* = 0.08, *p*‐value = .01) had larger impact on covariance in fragments than CN ratio (*β* = −0.11, *SE* = 0.06, *p*‐value = .04), with SM‐MH covariance decreasing in wetter, warmer sites (higher values of precipitation and temperature gradients; Figure 5a,b; Table S3). As with trait–abundance associations, models with elevation alone explained as much variation in trait covariance as models with multiple climate variables (see AIC and adjusted *R* ^2^, Table S4), except for SS‐MH covariance. However, all relationships with elevation were non‐significant.

## 4 DISCUSSION

In a human‐modified forest landscape, trait‐based assembly of tree communities across similar climate gradients differed in fragments compared to contiguous forests. However, trait–climate interactions alone (without random effects of species) explained negligible variation in species abundances across the landscape, suggesting other unmeasured factors about species that contributed to their distribution patterns. Net compositional change in contiguous forests occurred via multiple climate factors acting simultaneously to select combinations of traits, with elevation being a prominent gradient of assembly. Traits did not explain compositional change across fragments. Climate influenced site‐level covariance of seed size–maximum height, which showed that similar climatic conditions in fragments and contiguous forests favored different phenotypes.

The weak influence of any single trait on species' abundances indicated that assembly occurred along multiple climate axes selecting for multi‐trait phenotypes rather than any single trait, sometimes in opposing directions (Díaz et al., 2015; Shen et al., 2019). As a result, no combination of a single trait with a single macroclimate gradient explained the net compositional change in either fragments or contiguous forests. In contiguous forests, a composite gradient of higher elevation, greater soil C:N, and lower temperatures had a higher abundance of species with lower SLA and smaller seeds. This compositional pattern matched with trait–abundance associations, wherein abundances of larger‐seeded species decreased at higher elevations.

In addition, species abundances across the elevation gradient varied with maximum height and wood density. Given that only seed size played a role in mediating net compositional change, the abundance patterns for maximum height likely followed from its strong positive correlation with seed size. Wood density, however, comprised an independent trait axis (see traits PCA). The decrease in the abundance of denser‐wooded species at higher elevations may be due to unmeasured factors such as higher solar radiation that favor faster‐growing, low‐wood‐density species. Overall, patterns here suggest that trait‐mediated performance partly underlies the marked variation in species composition seen across the elevation gradient in contiguous forests (Krishnadas & Osuri, 2020), although the mechanisms remain unclear.

In fragments, macroclimate variables played a larger role than elevation in explaining trait–abundance associations, most prominently for seed size. Increased abundance of smaller‐seeded species at warmer sites may underlie their decrease at higher elevations where the climate was cooler than in lower elevations. Nonetheless, trait‐mediated changes in abundances did not translate to changes in functional composition across fragments. This may reflect the combined influence of fragment locations and shifts in species composition at 800 m ASL in the Western Ghats. Contiguous forests started at 630 m ASL, whereas fragments occurred only above 850 m ASL. Being restricted to higher elevations, fragments would experience less site‐to‐site variation in composition than contiguous forests. Thus, the loss of low‐elevation forests—typical of land‐use change—may have homogenized the tree community that remained across the landscape (Krishnadas & Osuri, 2020). This homogenization may continue if fragments favor the recruitment of some functional types regardless of elevation and climate (Frishkoff et al., 2019; Yano et al., 2021). Fragments may also experience more stochastic assembly or changes to local biotic interactions (Krishnadas, Bagchi, et al., 2018; Krishnadas & Osuri, 2020; Laurance, 2002), which may further override trait–climate linkages.

Selection for multi‐trait phenotypes across composite climate gradients may explain the relationships with individual climate variables that were contrary to expectation. For instance, dry conditions were expected to favor shorter‐statured species (Méndez‐Toribio et al., 2020), but taller species became more abundant as seasonal water deficit increased in contiguous forests. This may follow from seasonal water deficit decreasing at higher elevations where there was strong selection for species with shorter stature and smaller seeds. Another possibility is that even the greatest seasonal water deficit in this wet landscape did not impose significant drought stress or limit water transport, thus imposing minimal costs on taller species. Instead, fuller canopies in less seasonal sites may increase the abundances of shade‐tolerant understory species with shorter stature because light limitation poses the key stress to constrain trait combinations. This possibility was supported by the finding that water‐stressed sites had more flexible combinations of seed size and maximum height. An accurate understanding of the role of water deficit in community assembly would require measuring hydraulic traits that directly represent plant–water relations.

The weak role of individual traits in explaining compositional change also stemmed from the selection of trait combinations for seed size and maximum height (plot‐level SS‐MH covariance) across climate and elevation gradients. On average, fragments had stronger positive SS‐MH covariance than continuous forests, even as larger‐seeded species declined in fragments (Osuri & Sankaran, 2016a), which may be accounted for by two possibilities. The first possibility is the loss of mid‐canopy shade‐tolerant species with large seeds but shorter stature (Rüger et al., 2018), perhaps driven by water deficit. Shade‐tolerant, mid‐canopy species can be drought sensitive (Kupers et al., 2019; Sterck et al., 2011), and their loss may explain why sites with greater water stress had stronger positive SM‐MH covariance in fragments, even though we expect water‐stressed conditions to favor shorter species. Secondly, smaller‐seeded species were more abundant in warmer fragments, but species had taller stature for given seed size. The small seeds, tall stature phenotype is consistent with long‐lived pioneers (Rüger et al., 2018) and maybe a response to increased light availability in fragments.

## 5 CONCLUSION

Forest loss altered the effects of macroclimate on community assembly of trees and assembly in fragments occurred primarily via constraints on trait combinations. That seed size played a prominent role in assembly across climate gradients suggests that the dynamics of younger life stages (Krishnadas et al., 2020; Krishnadas, Bagchi, et al., 2018; Krishnadas & Comita, 2018; Larson et al., 2016), where seed size has a clear mechanistic influence on performance, may be crucial to future community structure in fragments (Bruun & ten Brink, 2008; Lebrija‐Trejos et al., 2016; Moles et al., 2014; Moles & Westoby, 2004; Visser et al., 2016). Overall, covariance patterns suggest that similar climate conditions select for different phenotypes in fragments and contiguous forests. Shifts in the success of phenotypes with forest loss could be due to edge effects that alter forest microclimates (Arroyo‐Rodríguez et al., 2017; Davis et al., 2019). Warmer and drier microclimates in fragments may amplify water stress during the dry season or exacerbate the effects of drier years in a changing climate via abiotic stress or by altering biotic interactions. Disentangling these processes requires mechanistic approaches targeted at traits involved in specific functions, e.g., water use or temperature tolerance. Uncovering the mechanistic basis of compositional change in fragments would improve our ability to manage and restore human‐modified landscapes.

## AUTHOR CONTRIBUTION

**Meghna Krishnadas:** Conceptualization (lead); data curation (lead); formal analysis (lead); methodology (lead); project administration (lead); resources (lead); supervision (lead); writing – original draft (lead); writing – review and editing (lead).

## FUNDING INFORMATION

This research did not receive any specific grant from funding agencies in the public, commercial, or not‐for‐profit sectors.

## Supporting information


Appendix S1

## Data Availability

The data used in this study can be publicly accessed from Dryad Digital Repository (https://doi.org/10.5061/dryad.7s7r1) and Supporting Information in Osuri and Sankaran (2016b).
