# Supplementary material for: Climate and forest loss interactively restructure trait composition across a human‐modified landscape
Source: Ecol Evol. 2022 Oct 30;12(11):e9361. doi: 10.1002/ece3.9361 (PMC9618670; doi:10.1002/ece3.9361)
Supplement: Supplementary file 1 — Appendix S1 [file ECE3-12-e9361-s001.docx]

**SUPPORTING INFORMATION: APPENDIX 1**

**Table S1.** Comparative model performance for climate variables vs. elevation in explaining trait-abundance associations. Separate models were implemented per trait and for fragments and contiguous forest.

|  |  | | Conditional R^2^ | | Marginal R^2^ | | AIC | |
| --- | --- | --- | --- | --- | --- | --- | --- | --- |
|  | |  | Climate | Elevation | Climate | Elevation | Climate | Elevation |
| Contiguous | SLA | | 0.40 | 0.41 | 0.02 | 0.01 | 5012 | 5012 |
| forest | Max.height | | 0.37 | 0.38 | 0.03 | 0.02 | 4981 | 4997 |
|  | Wood density | | 0.39 | 0.42 | 0.11 | 0.11 | 5012 | 5003 |
|  | Seed size | | 0.41 | 0.41 | 0.05 | 0.05 | 5020 | 5018 |
| Fragments | SLA | | 0.39 | 0.41 | 0.02 | 0.01 | 3600 | 3611 |
|  | Max.height | | 0.42 | 0.42 | 0.06 | 0.04 | 3606 | 3616 |
|  | Wood density | | 0.39 | 0.39 | 0.02 | 0.01 | 3619 | 3622 |
|  | Seed size | | 0.42 | 0.41 | 0.07 | 0.04 | 3608 | 3615 |

**Table S2.** Parameter estimates from models to assess how species abundances relate to the interaction of traits and elevation in fragments and contiguous forest. The analysis used generalized linear mixed effects model with negative binomial errors and zero-inflation component, with random intercepts for sites and species. Values in bold indicate significant effects. Numbers in parentheses are standard errors associated with each coefficient estimate.

| Habitat | Coefficient | SLA | Max. height | Wood density | Seed size |
| --- | --- | --- | --- | --- | --- |
| Contiguous forest | Intercept | 0.03 (0.2) | -0.03 (0.2) | -0.08 (0.19) | -0.05 (0.2) |
|  | Elevation | 0.09 (0.07) | 0.11 (0.06) | 0.12 (0.06) | 0.1 (0.07) |
|  | Trait | 0.15 (0.19) | -0.32 (0.19) | **0.74 (0.17)** | **0.44 (0.19)** |
|  | Elevation x trait | -0.09 (0.05) | **-0.16 (0.04)** | **-0.14 (0.05)** | **-0.16 (0.05)** |
| Fragments | Intercept | -0.01 (0.26) | -0.14 (0.26) | -0.2 (0.26) | -0.27 (0.25) |
|  | Elevation | 0.08 (0.19) | 0.11 (0.19) | 0.09 (0.19) | 0.16 (0.17) |
|  | Trait | -0.05 (0.25) | **-0.60 (0.24)** | 0.14 (0.24) | **-0.64 (0.23)** |
|  | Elevation x trait | -0.09 (0.13) | 0.01 (0.12) | -0.06 (0.12) | **0.40 (0.12)** |

**Table S3.** Parameter estimates for models testing trait covariance in relation to macroclimate gradients. Relationships were tested using linear mixed effects models with Gaussian errors. Plots nested within sites were included as random intercepts.

|  | **SLA-WD** | **SLA-MH** | **SLA-SS** | **WD-MH** | **WD-SS** | **SS-MH** |
| --- | --- | --- | --- | --- | --- | --- |
| (Intercept) | 0.35 (0.24) | 0.24 (0.31) | -0.03 (0.32) | 0.24 (0.31) | -0.03 (0.32) | **-0.04 (0.2)** |
| CN | -0.03 (0.02) | -0.06 (0.03) | -0.04 (0.03) | -0.06 (0.03) | -0.04 (0.03) | **0.05 (0.02)** |
| Climate PC1 | -0.01 (0.02) | 0.01 (0.03) | -0.01 (0.03) | 0.01 (0.03) | -0.01 (0.03) | -0.01 (0.02) |
| Climate PC2 | 0.04 (0.03) | 0.08 (0.04) | 0.04 (0.04) | 0.08 (0.04) | 0.04 (0.04) | **-0.06 (0.02)** |
| Climate PC3 | -0.06 (0.03) | -0.09 (0.04) | -0.05 (0.04) | -0.09 (0.04) | -0.05 (0.04) | **0.09 (0.03)** |
| Fragment | -0.66 (0.66) | -0.06 (0.86) | -0.14 (0.89) | -0.06 (0.86) | -0.14 (0.89) | **1.32 (0.55)** |
| SCN: Fragment | 0.07 (0.07) | -0.01 (0.09) | 0.01 (0.09) | -0.01 (0.09) | 0.01 (0.09) | **-0.12 (0.06)** |
| Clim.PC1: Fragment | 0.03 (0.03) | -0.05 (0.04) | -0.01 (0.04) | -0.05 (0.04) | -0.01 (0.04) | 0.02 (0.02) |
| Clim.PC2: Fragment | -0.11 (0.11) | -0.14 (0.14) | -0.22 (0.14) | -0.14 (0.14) | -0.22 (0.14) | **0.23 (0.09)** |
| Clim.PC3: Fragment | 0.06 (0.08) | -0.12 (0.1) | -0.14 (0.1) | -0.12 (0.1) | -0.14 (0.1) | **-0.15 (0.06)** |
| Adjusted R^2^ | 0.01 | 0.33 | 0.06 | 0.33 | 0.06 | 0.46 |


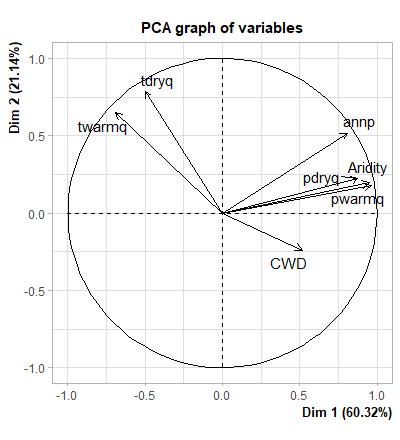


**Figure S1.** Principal Component Analysis (PCA) of precipitation and temperature variables from WorldClim. Seven variables were chosen to represent composite climate axes: mean annual precipitation (annp), precipitation of driest quarter (pdryq), precipitation of warmest quarter (pwarmq), temperature of driest quarter (tdryq), temperature of warmest quarter (twarmq), climatic water deficit (CWD), and climate aridity.


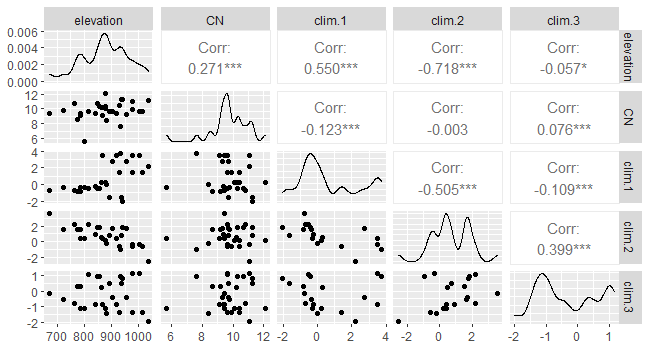


**Figure S2. Correlations among climate variables in contiguous forest**. CN = soil C:N ratio, clim 1, clim 2, and clim 3 are composite axes from PCA of multiple climate variables (see Figure S1).


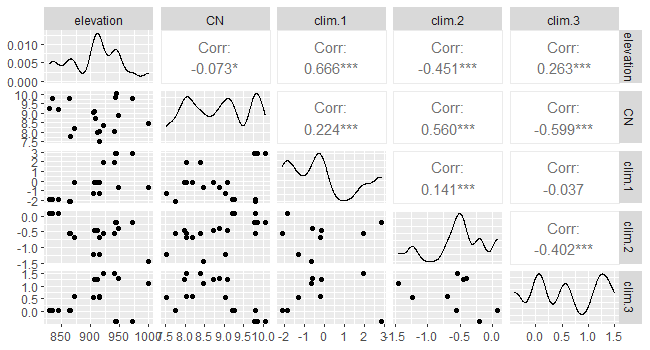


**Figure S3. Correlations among climate variables across forest fragments.** CN = soil C:N ratio, clim 1, clim 2, and clim 3 are composite axes from PCA of multiple climate variables (see Figure S1).


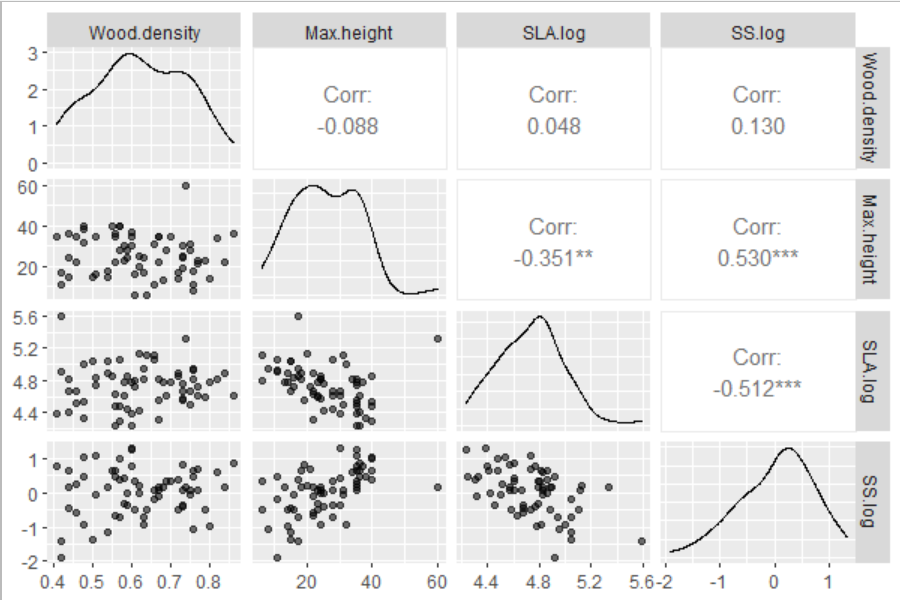


**Figure S4. Pairwise correlation of traits.** Pearson coefficients of correlation between each pair of the four traits used in the study.
